# Laparoscopically assisted distal gastrectomy with Billroth I anastomosis for refractory gastric outlet obstruction after endoscopic submucosal dissection procedure: a case report

**DOI:** 10.1093/jscr/rjaf946

**Published:** 2025-12-04

**Authors:** Spartak Alexey, Ivashov Ivan, Pilat Tatyana, Xiaodong Lei, Tao Li, Kuznetsova Julia, Shcherbaniuk Ilona

**Affiliations:** Department of Faculty Surgery No. 2, Named after G.I. Lukomsky, Federal State Autonomous Educational Institution of Higher Education I.M. Sechenov First Moscow State Medical University of the Ministry of Health of the Russian Federation (Sechenov University), 8-2 Trubetskaya Str., Moscow 119991, Russia; Department of Faculty Surgery No. 2, Named after G.I. Lukomsky, Federal State Autonomous Educational Institution of Higher Education I.M. Sechenov First Moscow State Medical University of the Ministry of Health of the Russian Federation (Sechenov University), 8-2 Trubetskaya Str., Moscow 119991, Russia; The Federal State Budgetary Scientific Institution “Izmerov Research Institute of Occupational Health”, 31, Prospect Budennogo, Moscow 105275, Russia; School of Medical and Life Sciences, Chengdu University of Traditional Chinese Medicine, 37 Shierqiao Road, Jinniu District, Chengdu, Sichuan 610075, China; School of Medical and Life Sciences, Chengdu University of Traditional Chinese Medicine, 37 Shierqiao Road, Jinniu District, Chengdu, Sichuan 610075, China; Department of Gastroenterology, Central State Medical Academy of the Administrative Directorate of the President of the Russian Federation, 19 Marshal Timoshenko str. 1A, Moscow 121359, Russia; Department of Faculty Surgery No. 2, Named after G.I. Lukomsky, Federal State Autonomous Educational Institution of Higher Education I.M. Sechenov First Moscow State Medical University of the Ministry of Health of the Russian Federation (Sechenov University), 8-2 Trubetskaya Str., Moscow 119991, Russia

**Keywords:** endoscopic submucosal dissection, gastric outlet obstruction, gastroptosis, surgical treatment

## Abstract

Endoscopic submucosal dissection (ESD) has become the standard minimally invasive treatment for early gastric cancer and precancerous lesions. However, performing extensive resections near the pyloric ring or antrum may lead to post-ESD gastric outlet obstruction (GOO) due to scar-related stenosis and luminal deformation. This report presents a case of a 62-year-old female patient who developed severe GOO after ESD treatment for a poorly differentiated antral adenocarcinoma (pT1aN0M0). Initial interventions involved two endoscopic balloon dilations at the pyloric stricture, which failed to provide lasting relief of symptoms. Subsequently, a salvage treatment was performed with laparoscopic-assisted distal gastrectomy with Billroth I reconstruction, resulting in complete resolution of obstruction symptoms and restoration of normal oral intake. This case highlights the challenges in managing post-ESD GOO and demonstrates that laparoscopic-assisted distal gastrectomy with Billroth I reconstruction is an effective salvage option for refractory cases. Further studies are needed to optimize treatment strategies and evaluate long-term outcomes.

## Introduction

Endoscopic submucosal dissection (ESD) is an endoscopic technique that removes the mucosa of lesions using various specialized high-frequency electric knives to achieve therapeutic goals [1]. It has been widely used for the treatment of early gastrointestinal tumors. Narrow luminal areas, such as the esophagus, gastroesophageal junction, or lesions located in the pyloric region, are high-risk factors for stricture after ESD [2]. Gastric mucosal resection within 0.5 cm of the pyloric ring is defined as pyloric ESD. Pyloric stricture after ESD refers to the inability of a gastroscope with a diameter of 9.2–10.2 mm to pass through the pyloric ring, preventing access to the duodenum, and presents as pyloric deformation, narrowing of the pyloric orifice, or both [1].

The incidence of stenosis after gastric ESD is 1.6% ~ 2.5%, whereas the incidence of stenosis after pyloric ESD is higher, at 3.1% ~ 16% [1]. After ESD of lesions located in the pyloric region, an “artificial ulcer” is formed. Contraction of the muscularis propria leads to ulcer shrinkage and pyloric stenosis during scar healing, which is related to factors such as scar constitution, circumferential range of the pylorus, length of lesion resection in the prepyloric area, and the presence of antrum or pyloric deformities. High-risk factors for pyloric stenosis are ESD resection exceeding or reaching three-quarters of the pyloric mucosa, and are not related to the specific location of pyloric ESD, the presence of ulcers, histopathological type of the excised lesion, or the depth of the lesion. The incidence of pyloric stenosis when reaching or exceeding three-quarters of the pyloric circumference is 84% ~ 88.9%, and the incidence of stenosis after circumferential pyloric ESD is 100%. A lesion with a long diameter >5 cm in the prepyloric area is a risk factor for pyloric stenosis after pyloric ESD [3]. In cases of antral or pyloric deformities, even if ESD excision of the pyloric circumferential mucosa is less than three-quarters and the longitudinal resection range of the lesion is <5 cm, pyloric stenosis is still prone to occur after ESD [4].

Postoperative ESD stenosis may cause gastric outlet obstruction (GOO) due to submucosal fibrosis and scar formation, leading to difficulty in food passage and adversely affecting the patients’ nutritional status and quality of life [1]. Gastric antral stenosis can be treated with endoscopic balloon dilation (EBD), endoscopic antralplasty, self-expandable metal stents (SEMS), and other endoscopic treatments [5, 6]. However, if the stenosis caused by deformity is severe and recurs despite the above treatments, other therapeutic strategies are required.

## Case presentation

A 62-year-old female patient was admitted for surgical resection after routine examination revealed moderately differentiated adenocarcinoma of the gastric antrum. The patient had concomitant hypothyroidism and grade II-III gastroptosis.

On 26 December 2024, Endoscopic surgery was performed: the tumor was resected endoscopically via ESD in the abnormal mucosal pattern areas of the gastric antrum and the lower third of the gastric body, measuring ~6 × 3 cm, leaving a defect covering three-quarters of the circumference of the gastric antrum. Histological diagnosis: poorly differentiated adenocarcinoma pT1aN0M0, LV- Pn- (HM0, VM0). Early postoperative period involved vomiting of blood and gastric content retention. During follow-up gastroscopy, pyloric incomplete stenosis was diagnosed. On 4 and 6 February 2025, EBD for the pyloric stenosis was performed, but symptom relief was minimal.

**Figure 1:**
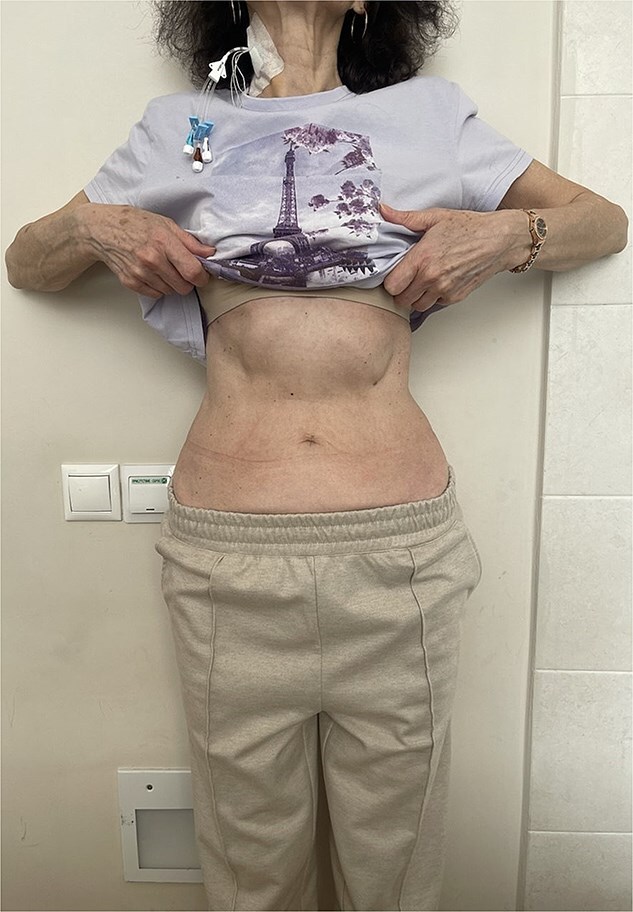
Clinical photograph of the patient’s abdomen at admission.

**Figure 2:**
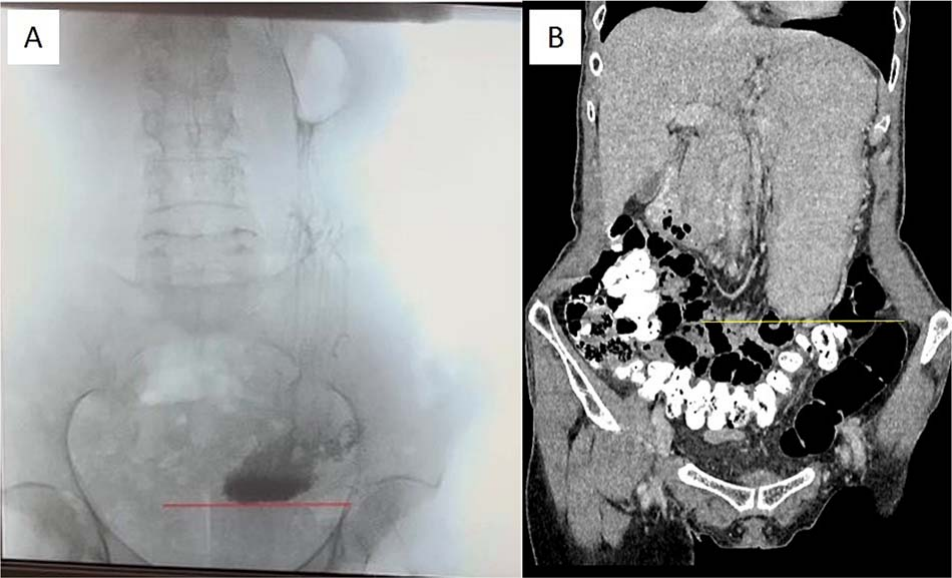
Pre-operative imaging confirming GOO. (A) X-ray of the stomach and duodenum with contrast medium. Line indicates the lower border of the stomach. (B) CT scan of the abdomen with intravenous contrast. Line demarcates the lower border of the stomach.

**Figure 3:**
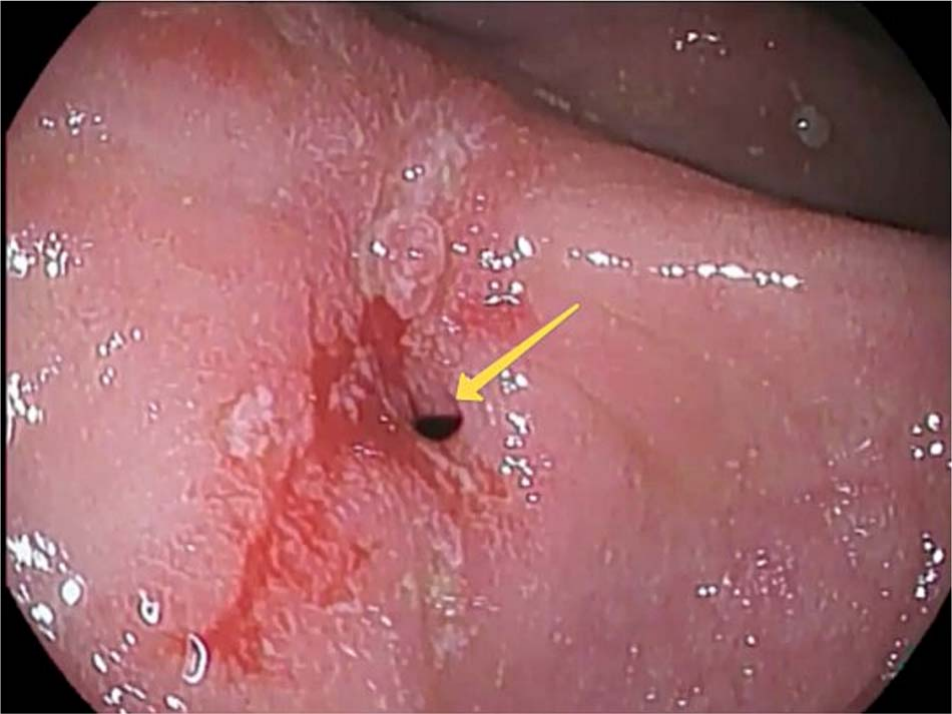
Pre-operative esophagogastroduodenoscopy (EGD) view. An arrow indicates the pylorostenosis.

At this admission, the patient reported feeling “Bloated in the stomach, unable to eat solid foods, belching, bitter taste in the mouth, and weight loss (7 kg). BMI was 16.1 upon admission and showed signs of malnutrition” (Fig. 1). Gastric and duodenal radiography on 1 April 2025 showed the distal stomach located in the pelvic projection range, and the stomach had not emptied after observation for 2 hours (Fig. 2A). Combined X-ray and computed tomography (CT) findings revealed severe gastroptosis (Fig. 2B), and repeat endoscopy indicated pyloric obstruction, with difficulty passing the endoscope through the pyloric ring. Ulcer stenosis at the ESD site led to deformation of the gastric antrum and GOO, with chronic food retention and continuous gastric acid stimulation causing scar healing (Fig. 3). Given the extensive stenosis, EBD treatment was deemed likely ineffective. Prior to surgery, and to compensate for the nutritional deficiency, the patient received enteral nutrition with “Leovit ONCO” according to the clinic’s protocol, and laparoscopic-assisted distal gastrectomy with Billroth I anastomosis was planned.

**Figure 4:**
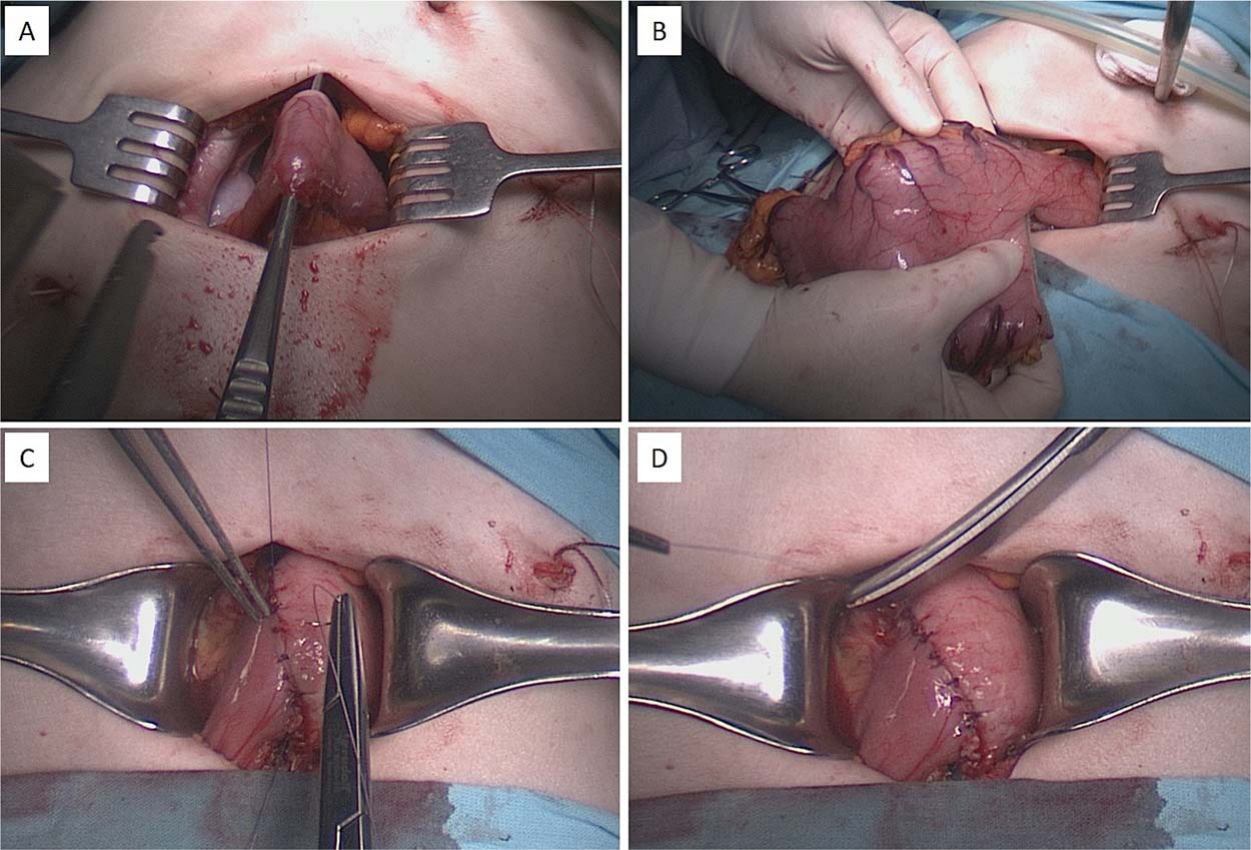
Intraoperative procedure of laparoscopically assisted distal gastrectomy with Billroth I anastomosis. (A and B) Extraction and transection of the distal stomach through a mini-laparotomy access wound. (C and D) Creation of a hand-sewn, double-layered gastroduodenal (Billroth I) anastomosis.

**Figure 5:**
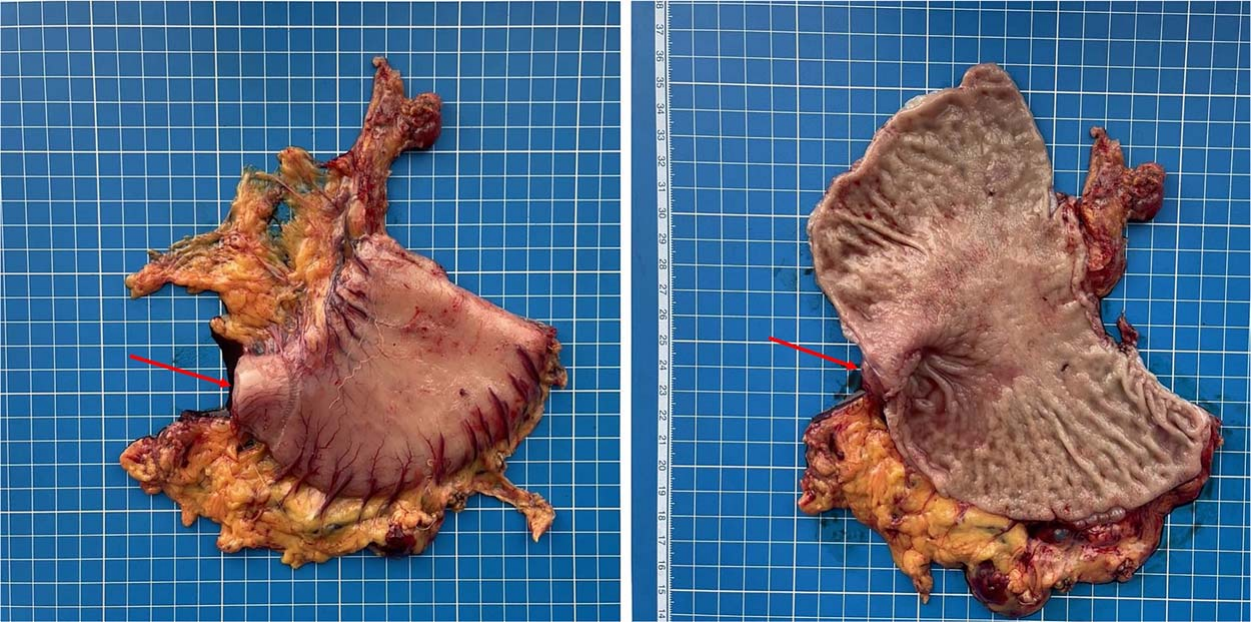
Gross specimen of the resected distal stomach. The arrows indicate the area of severe fibrotic stenosis in the pyloroduodenal region.

**Figure 6:**
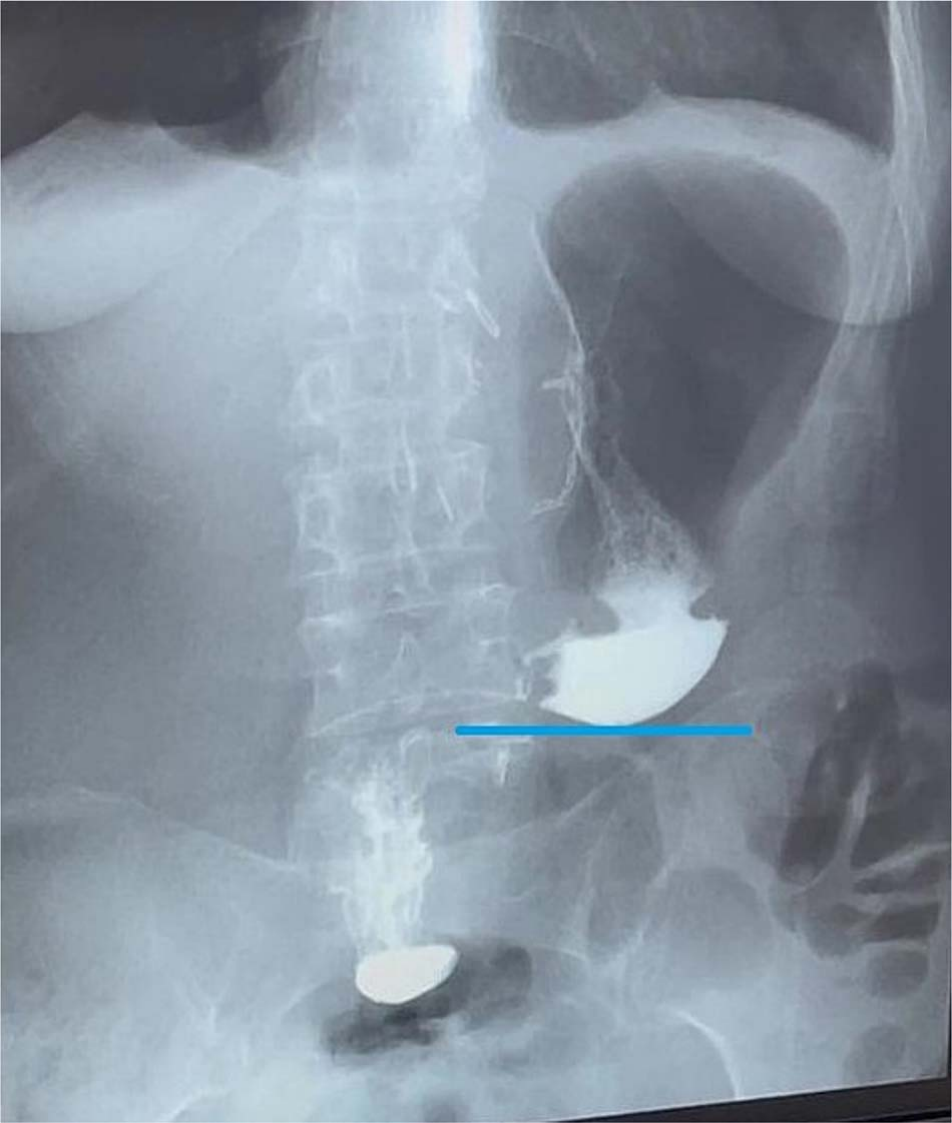
Contrast X-ray study performed on the third postoperative day. The image demonstrates free passage of contrast through a widely patent gastroduodenal (Billroth I) anastomosis without evidence of leakage or obstruction. Line indicates the newly configured lower border of the gastric remnant.

On 10 April 2025, laparoscopically-assisted distal subtotal gastric resection with Billroth I anastomosis was performed. Intraoperatively, a long, sagging stomach reaching the pelvic level was observed, and after laparoscopic gastric mobilization, a hand-sewn interrupted double row anastomosis was formed using a minilaparotomy approach (Fig. 4).

Final surgical pathology report showed scarring deformation of the walls of the antrum of the stomach with signs of chronic inflammation in the muscular coat. No tumor growth was detected (Fig. 5). In 40 examined lymph nodes, there was no tumor growth. The postoperative period was uneventful. On the 3rd day after the operation, a control X-ray examination was performed using a water-soluble contrast agent. It showed that gastric evacuation was timely, and no contrast agent leakage was detected (Fig. 6). The patient was discharged on 7th day after operation with oral nutrition fully restored*.*

## Discussion

Due to the anatomical characteristics of the stomach, the treatment of gastric antrum stenosis after ESD is difficult. If the stenosis persists, it can lead to GOO, seriously affecting the patient’s nutritional intake [3]. The main risk factors for stenosis formation include the extent of mucosal resection, the degree of submucosal injury, and whether there is bleeding or infection [1, 7].

EBD is currently the most widely used method for treating gastric antral or pyloric stenosis after ESD. However, for benign pyloric stenosis, the treatment success rate with EBD is not ideal, with only 16%–70% of patients experiencing sustained symptom relief, 2.8%–4.3% requiring emergency surgery due to perforation, and 22%–32% needing repeated treatments [8, 9]. For some patients, endoscopic antralplasty can also be considered. However, the direction of traction on the scarred stenosis under endoscopy is difficult to predict. In cases of severe gastric antrum deformation, it cannot effectively prevent restenosis. Sometimes local steroid injections are administered after incision to prevent restenosis, but steroid use carries the risk of delayed perforation or infection. Additionally, partially covered SEMS can be implanted. However, issues such as stent migration and the optimal duration for stent placement remain unresolved [10]. Table 1 shows the treatments of refractory GOO after ESD. Therefore, when the aforementioned treatments are ineffective, performing a distal subtotal gastric resection with Billroth I anastomosis is a safe and effective treatment method [11].

**Table 1:** Comparison of treatment modalities for refractory GOO after ESD

	Effectiveness	Number of operations	Risk of restenosis	Complication	Applicable population
EBD	High (85.6%–97.3%)	Multiple times (average 3–7 times)	22%–32% require repeated expansion	Perforation rate is about 3.8%, bleeding is about 3.4%	First-line treatment
SEMS	Medium (81.8% to nearly 100%)	Once	33.3% recurrence rate within 6 months	62.5% experienced stent displacement, infection (rare)	Remedial measures after EBD failure
Local steroid therapy	Ineffective in preventing restenosis	1–2 times			Early intervention
Endoscopic pyloroplasty	Medium to high (87% to nearly 100%)	Multiple times	37.5% recurrence rate within 6 months	Perforation (rare) bleeding (12.5%)	EBD/bracket invalid
Surgical treatment	High (nearly 100%)	Once	Recurrence rate <10%	Mild to moderate complications ≈ 10%–20% Severe complications <5% Reoperation rate <3%	The definitive treatment after the failure of endoscopic methods

ESD, endoscopic submucosal dissection; EBD, endoscopic balloon dilation; SEMS, self-expandable metallic stent.

The patient had a rare condition of grade II–III gastroptosis combined with pyloric stenosis, and the combination significantly exacerbates gastric emptying disorders; due to gastroptosis, the stomach is positioned too low, causing gastric peristalsis to work against gravity, which delays gastric emptying; pyloric stenosis further obstructs the discharge of gastric contents, forming a vicious cycle of gastric retention, reduced gastric tension, worsening gastroptosis [12]. Treatments such as endoscopy and medication have limited effects, necessitating further surgical intervention. Therefore, to prevent complications related to gastroptosis, such as gastric emptying disorders, stenosis recurrence, and impaired nutrient absorption, we chose to perform a partial gastrectomy on this patient.

## Conclusion

For post-ESD pyloric stenosis, in cases where conventional endoscopic dilation and pyloroplasty fail, especially in cases of recurrent stenosis or moderate to severe gastric ptosis, Surgical treatment can be considered an effective treatment option. Future research should further analyze the latest treatment methods for pyloric obstruction and explore adjuvant therapies to prevent stenosis recurrence. In addition, it is necessary to study the long-term efficacy of surgery as a treatment for pyloric stricture after ESD and to develop strategies to reduce surgery-related complications.
